# A Neonatal Mouse Spinal Cord Injury Model for Assessing Post-Injury Adaptive Plasticity and Human Stem Cell Integration

**DOI:** 10.1371/journal.pone.0071701

**Published:** 2013-08-19

**Authors:** Jean-Luc Boulland, François M. Lambert, Mark Züchner, Susanne Ström, Joel C. Glover

**Affiliations:** 1 Laboratory of Neural Development and Optical Recording (NDEVOR), Department of Physiology, Institute of Basic Medical Sciences, Faculty of Medicine, University of Oslo, Oslo, Norway; 2 Norwegian Center for Stem Cell Research, Department of Immunology and Transfusion Medicine, Oslo University Hospital, Oslo, Norway; 3 Department of Neurosurgery, Oslo University Hospital, Oslo, Norway; Hospital Nacional de Parapléjicos - SESCAM, Spain

## Abstract

Despite limited regeneration capacity, partial injuries to the adult mammalian spinal cord can elicit variable degrees of functional recovery, mediated at least in part by reorganization of neuronal circuitry. Underlying mechanisms are believed to include synaptic plasticity and collateral sprouting of spared axons. Because plasticity is higher in young animals, we developed a spinal cord compression (SCC) injury model in the neonatal mouse to gain insight into the potential for reorganization during early life. The model provides a platform for high-throughput assessment of functional synaptic connectivity that is also suitable for testing the functional integration of human stem and progenitor cell-derived neurons being considered for clinical cell replacement strategies. SCC was generated at T9–T11 and functional recovery was assessed using an integrated approach including video kinematics, histology, tract tracing, electrophysiology, and high-throughput optical recording of descending inputs to identified spinal neurons. Dramatic degeneration of axons and synaptic contacts was evident within 24 hours of SCC, and loss of neurons in the injured segment was evident for at least a month thereafter. Initial hindlimb paralysis was paralleled by a loss of descending inputs to lumbar motoneurons. Within 4 days of SCC and progressively thereafter, hindlimb motility began to be restored and descending inputs reappeared, but with examples of atypical synaptic connections indicating a reorganization of circuitry. One to two weeks after SCC, hindlimb motility approached sham control levels, and weight-bearing locomotion was virtually indistinguishable in SCC and sham control mice. Genetically labeled human fetal neural progenitor cells injected into the injured spinal cord survived for at least a month, integrated into the host tissue and began to differentiate morphologically. This integrative neonatal mouse model provides opportunities to explore early adaptive plasticity mechanisms underlying functional recovery as well as the capacity for human stem cell-derived neurons to integrate functionally into spinal circuits.

## Introduction

Adaptive plasticity in the spinal cord has become an important focus in spinal cord injury (SCI) research due to increasing evidence that spinal networks in the injured spinal cord of rodents and cats can reorganize spontaneously following an injury [1]–[8], and that this reorganization can be promoted by experimental manipulation [2], [3], [9]–[12]. Post-injury plasticity in the spinal cord involves the sprouting of spared axons and the formation of novel synaptic connections and may or may not promote functional recovery, depending on whether it is adaptive or maladaptive [13]. The potential benefits of adaptive plasticity are fueling efforts to introduce plasticity-augmenting procedures into clinical trials on human SCI patients [14].

The adaptive plasticity exhibited by the adult brain and spinal cord in connection with learning, memory and recovery from injury is believed to involve at least in part the same mechanisms that underlie the plasticity of the developing nervous system [15], [16]. Insight into the mechanisms governing adaptive plasticity following injury in the adult spinal cord may therefore be gained by characterizing adaptive plasticity following injury in the immature spinal cord.

SCI also occurs in infants and children in a variety of scenarios, including birth trauma, accidents, and non-traumatic causes. Pediatric SCI represents on the order of 5% of all SCI cases but may be underreported, and is certainly less well investigated than adult SCI [17]. A particular injury category that is virtually exclusive to the pediatric population is ‘spinal cord injury without radiographic abnormality’ (SCIWORA) [18]. SCIWORA is a consequence of the highly elastic properties of the immature spine, which permits stretching of the spinal column to extents that cause injury to the spinal cord with no overt injury to skeletal structures. Very little is known about the pathogenetic processes involved in pediatric SCI and its potential recovery, providing an additional incentive to investigate mechanisms of adaptive plasticity in the immature spinal cord.

Only a few studies have investigated recovery after SCI that has been generated in newborn rodents. Some have focused, as in adult studies, on behavior and anatomy [19], [20], whereas others have begun to assess the molecular and cellular substrates of recovery, either soon after the injury, at early postnatal stages [21] or much later, in the adult [22]–[24]. The neonatal mouse spinal cord has become a popular preparation for the study of the normal spinal cord circuitry involved in locomotor pattern generation [25]–[27] and in the descending control of spinal motor and autonomic circuits [25], [28]–[32]. The neonatal mouse is amenable to a combination of molecular, genetic, anatomical, physiological, and behavioral approaches to the elucidation of neuronal network organization. In particular, because of its small size and the possibility to use highly tractable *ex vivo* preparations, it is highly amenable to electrophysiological approaches [33], rapid and precise tract-tracing [34] and high-throughput optical recording approaches that permit the dynamic assessment of functional synaptic connections onto large numbers of spinal neurons simultaneously [29]–[32], [35], [36].

For these reasons, we have become interested in developing a neonatal mouse SCI model in which mechanisms underlying adaptive plasticity can be studied with an integrated platform of approaches, at high throughput and high cellular resolution. In this regard, we have had two main goals. The first has been to investigate adaptive plasticity exhibited by brain stem and propriospinal projections to spinal neurons. The second has been to develop a model system in which human stem and progenitor cells can be introduced into an injured spinal cord during the process of adaptive plasticity to investigate whether this promotes the functional synaptic integration of derivative human neurons into spinal networks.

Descending pathways from the brain stem to the spinal cord and propriospinal pathways are both of utmost importance for the control of locomotion, posture and autonomic function in mammals [37]–[39]. SCI can interrupt these pathways and thus impair limb control, interlimb coordination and cardiovascular, bowel, bladder and sexual functions to varying degrees [40]. It is therefore important to elucidate the extent to which reorganization in these pathways occurs after SCI and how this facilitates functional recovery. However, the pathways subserving various motor and autonomic functions are diverse. A key goal in this regard is therefore to assess the range of adaptive plasticity exhibited by specific descending tracts and propriospinal networks, and to determine whether augmenting plasticity can be targeted selectively so as to avoid non-specific effects that may be maladaptive.

Parallel to the recent advances in understanding adaptive plasticity in the spinal cord, stem cell-based strategies have been proposed for replacing neurons that are lost to SCI and other spinal cord disease (reviewed in [41]). The possibility of generating any specific type of spinal neuron from stem or progenitor cells and designing cell replacement therapies based on these has raised the hope of restoring spinal cord function even in cases when intrinsic adaptive plasticity may be insufficient. However, introducing new neurons into a damaged or diseased spinal cord and coaxing them to survive and integrate into synaptic circuits has its own set of hurdles and is also saddled with the problem of avoiding maladaptive sequelae. It is therefore crucial in this regard to design reproducible SCI models into which different types of human stem and progenitor cells can be transplanted and their functional integration tested.

Here we have developed a neonatal mouse SCI model in which behavioral recovery is substantial and relatively rapid, and in which we can readily study adaptive plasticity at multiple levels, from structure to physiology to behavior. We demonstrate much greater behavioral recovery after thoracic compression injuries than after complete thoracic transections, we use immunohistochemistry, electron microscopy and tract tracing to characterize the extent of damage after compression injury, and we use electrophysiological and high-throughput optical recording to characterize the recovery of functional synaptic connections from specific descending tracts onto specific subpopulations of lumbar motoneurons. We also show that human fetal neuronal progenitor cells transplanted into the compression-injured neonatal mouse spinal cord can survive for weeks, during which time they exhibit signs of morphological differentiation into neurons. We note that high-throughput optical recording can also be used to assess descending connections onto specific subpopulations of spinal interneurons [31] and onto transplanted human cells using either conventional or optogenetic activity probes. This integrative, multi-methodological platform employing the neonatal mouse should provide novel insight into the scope of adaptive plasticity in the developing mammalian spinal cord, at the level of specific neuronal network components, including new neurons introduced into the spinal cord through cell replacement therapies. We suggest that such information may also be relevant for understanding adaptive plasticity in the injured adult spinal cord.

## Materials and Methods

### Spinal Cord Compression Clip

**Figure 1:**
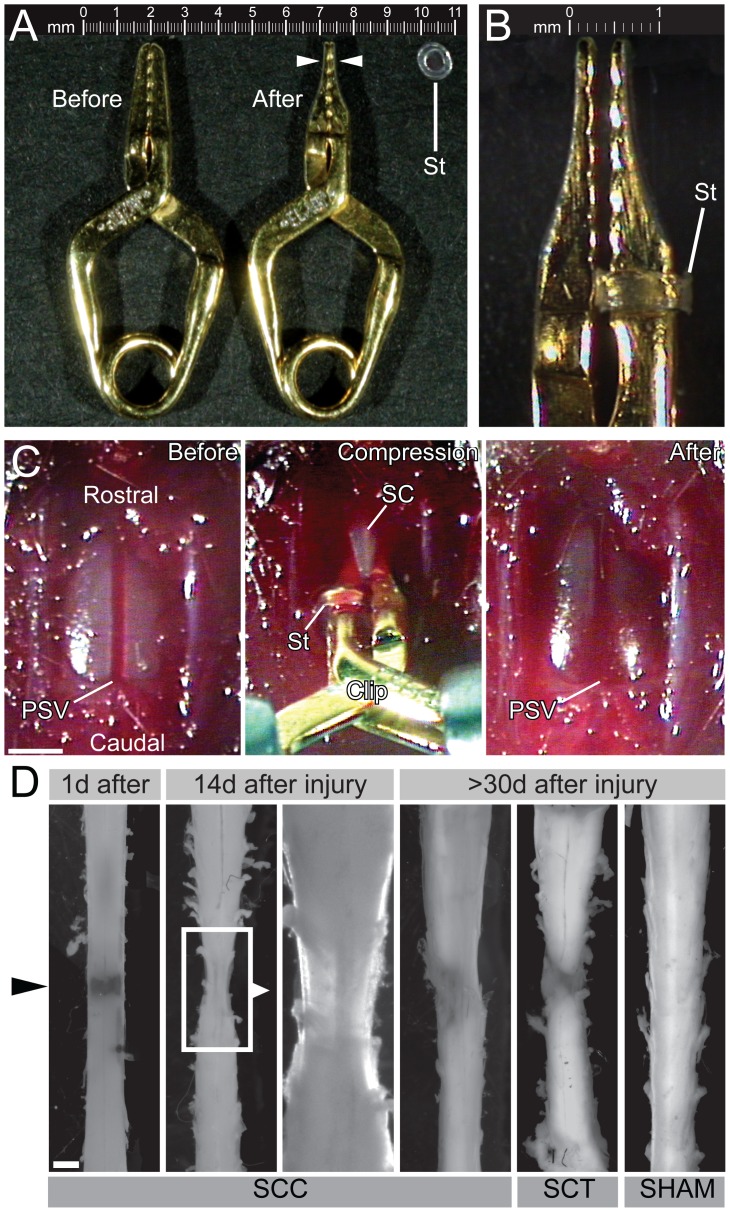
Modifications of the Yasargil aneurysm clip used for SCC, and tissue appearance following SCC. (A) Before (left) and after (right) trimming of the clip blades down to 150 µm. The stopper (St) was fashioned from a slice of polypropylene tubing. (B) When fully released, the clip maintains an interblade distance of 230 µm. (C) Image sequence from surgery showing the laminectomy (“Before”), the clip-driven compression, and the subsequent hemorrhagic contusion and edema (“After”). (D) Representative spinal cords dissected at different times after injury. The level of the compression site is indicated by the black arrowhead. The third panel is a high magnification view of the boxed area in the second panel. Scale bars: 1 mm.

A surgical aneurysm clip (Yasargil temporary aneurysm mini-clip, ref. FE681K; applicator from Æsculap, Center Valley, PA, USA) was modified so that it could be used to generate spinal cord compression (SCC) injuries in newborn mice. The outside edges of the blades of the clip were filed down so that blade thickness was 150 µm (Fig. 1A) and a stopper made from a polyethylene capillary tube (Polyethylene tubing, ID 0.58 mm, OD 0.96, Smiths Medical, UK, ref. 800/100/200) was placed on one of the blades to prevent full closure. The interblade distance at full closure was thus standardized to 230 µm (Fig. 1B).

### Spinal Cord Compression Injury

Most surgeries were performed on postnatal day (P)1 wild-type ICR mice (The Jackson Laboratory, Bar Harbor, ME, USA). For fetal neural progenitor cell injection experiments, immunocompromised ICR-SCID mice (Taconic, Denmark) were used to avoid immune rejection. The experimental protocol was approved by the Norwegian National Animal Research Authority (FELASA, local experimental approval number 12.4591) in compliance with European Union animal care regulations and the National Institutes of Health guidelines for the care and use of animals. Efforts were made to minimize the number of animals used and their suffering. Animals had free access to food and tap water and were maintained on a 12 hour light/dark cycle at 21±1°C. Anesthesia was initiated in a closed chamber using 4% isoflurane (Forene; Abbot-Norge, Norway) vaporized in pure oxygen using a Cyprane vaporizer (Keighley, UK). When deeply sedated, the mouse was placed onto a thermal blanket and the snout inserted into a mask providing a continuous supply of isoflurane and oxygen. Complete analgesia was obtained by subcutaneous injection of 50–100 µg of the local anesthetic Marcain (AstraZeneca Norge, Norway) at the surgery site (thoracic level (T)9-T12) allowing subsequent reduction of the isoflurane concentration to 1–2%. After a transverse skin incision and muscle dissection a laminectomy was performed at T9–12 using thin scissors and thin forceps (Fine Science Tools, Heidelberg, Germany). Leaving the dura intact, parts of the facet joints were removed to gain enough lateral space to insert the compression clip and an extradural spinal cord compression was performed (Fig. 1C). The spinal cord was compressed two times for 15 s each with reversal of the blades in between to achieve symmetrical compression. For complete spinal cord transection (SCT), the dura was pierced and opened using a flame-etched 100 µm diameter tungsten needle and the cord was cut transversely with fine scissors. To ensure complete transection, the inner vertebral walls were scraped in the plane of the transection with a micro-knife (Fine Science Tools) to cut all remaining fibers. Muscle and skin layers were sutured with coated Vicryl 6–0 (Johnson and Johnson, Berkshire, UK). After surgery was completed, mice received a subcutaneous injection of 50 µl of a nutrition-replacement solution (Pedamix; Fresenius Kabi, Homburg, Germany), removed from the anesthesia mask and placed in a temperature-controlled isolation cage for 2–3 hours. Before returning the mice to the litter, the mother was injected with diazepam (Stesolid, 8 g/kg body weight; Actavis, Iceland) to reduce aggressiveness and cannibalism. During the first postoperative days, 30 µl of buprenorphine (Temgesic, 0.03 mg/ml; Schering-Plough, Belgium) were injected subcutaneously once a day for pain relief. In case of bladder dysfunction (which was not typical following compression injury, but occurred after transection injury), bladder massage was performed twice a day until bladder function was restored. Apart from the injury itself, sham control animals were treated the same way as injured animals, including anesthesia, skin and muscle incision and laminectomy.

### Behavioral Analysis

A non-weightbearing air stepping test (modified from [42]) was used to quantify limb motility in sham control, SCC and SCT mice at different times after surgery/injury (Table 1, total n = 35). Tests were performed under a heating lamp to avoid hypothermia, especially at the earliest ages (1 and 4 days after surgery/injury). Animals were suspended dorsal up from a horizontal bar in a soft harness that secured the head, body and tail without restraining limb movements. The harness was adjusted to secure the animal so that there was no obvious suffering or discomfort. Typically, once restrained, the animal was quiescent and did not struggle unless stimulated. The paws were marked in black to facilitate video kinematic tracking. A mirror was mounted below the animal at a 45-degree angle to permit recording of side and ventral views simultaneously. After a short period of quiescence the nose was touched with a smooth metal prod, which led to a series of what appeared to be escape movements involving alternating flexion and extension of each limb (air stepping) with variable interlimb coordination. Video sequences of 1–3 min were taken with a 25 Hz camera (JVC Everio), and at the end of this session the animal was returned to the litter. Raw interlaced video images were reformatted into real images using the Avidemux video editor (http://www.avidemux.org/), sequences of these were imported into ImageJ [43] and the trajectory of each paw was tracked frame-by-frame using a “manual tracking” plug-in [44]. The trajectories (movements of the paws in the x-y plane), trajectory amplitudes (maximal distance traversed in the x-y plane) and instantaneous velocities (interframe distance covered divided by interframe duration) were measured.

**Table 1:** Summary of the number of mice used for each procedure performed at different timepoints after injury (SCC, SCT) or surgery (sham).

Procedure performed	Surgery/injury	Days after surgery/injury						
		≤1	4	8	14	24	≥29	
Wholemount observation	SHAM	>10			5		5	
	SCC	>10			6		6	
	SCT						6	
Retrograde labeling	SHAM	3			5			
	SCC	6			6			
Electron microscopy	SHAM	3						
	SCC	2						
Behavior (air-stepping)	SHAM	4	7	6	5			
	SCC	8	7	7	4			
	SCT		6	3	3			
Behavior (gait analysis)	SHAM					3		
	SCC					3		
Electrophysiology	SHAM	3	3	2				
	SCC	5	4	2				
Calcium imaging	SHAM	4	3					
	SCC	5	5					
Immunohistochemistry	SHAM	1		2	3			
	SCC	2			3			
Analysis of transplanted progenitor cells	SCC						5	

A single-track locomotion test was used to quantify gait parameters in sham control and SCC mice (n = 3 for each group) 24 days after surgery/injury. For gait analyses, mice were placed in a transparent, rectangular, ceilingless plexiglass corridor (10 cm wide×50 cm high). Mice placed at one end walked readily along the corridor following a gentle push from behind, and were videofilmed at 25 Hz through the floor of the corridor. A frame-by-frame analysis was performed as above and stance durations were determined by measuring the time from initial contact to final lift of each paw during successive placements.

### Ex vivo Brainstem-spinal Cord Preparation

The dissection procedure for obtaining *ex vivo* preparations of the isolated brain stem and spinal cord of neonatal mice has been described previously [28]–[31]. In brief, after deep anesthesia (isoflurane), rapid decerebration and evisceration, a posterior craniotomy and ventral laminectomy were performed in ice-cold (1–5°C), oxygenated (95% O2 and 5% CO2) glycerol-based dissecting solution (in mM: glycerol 250, KCl 2, D-glucose 11, CaCl2 0.15, MgSO4 2, NaH2PO4 1.2, HEPES 5, and NaHCO3 25) to expose the brain stem and the spinal cord with the VIIIth cranial nerves and ventral spinal roots intact. The brain stem and spinal cord were then removed into oxygenated artificial cerebrospinal fluid (ACSF, containing in mM: NaCl 128, KCl 3, D-glucose 11, CaCl2 2.5, MgSO4 1, NaH2PO4 1.2, HEPES 5, and NaHCO3 25). The same type of preparation was used for electrophysiology and optical recording experiments.

### Retrograde Labeling of Spinal Interneurons, Propriospinal Neurons and Projection Neurons

The procedure for labeling spinal interneurons, propriospinal neurons and projection neurons with conjugated dextran amine tracers has been described previously [34], [45], [46]. Briefly, after deep isoflurane anesthesia, the animal was decapitated and eviscerated, a complete ventral laminectomy was performed in dissecting solution to expose the entire spinal cord, and the preparation was transferred to oxygenated ACSF at room temperature (24–28°C). A transverse cut was made across the right ventral and ventrolateral funiculi, to about midway between the ventral and dorsal surfaces of the spinal cord, one segment below the compression site in spinal cord injured animals or at the same segmental level in sham controls. Immediately thereafter, several (5–7) small crystals of Rhodamine Dextran Amine (RDA; tetramethylrhodamine dextran, lysine fixable, 3000 MW; Invitrogen) were applied sequentially to the cut creating an exposure to a high concentration of the tracer for 3–5 minutes. The preparation was incubated for 6–7 hours at room temperature in oxygenated ACSF in the dark, after which an 8 segment long piece of the cord centered on the compression site or equivalent segment in sham controls was fixed overnight in 4% paraformaldehyde (PFA; TAAB Laboratories Equipment Ltd, Aldermaston, Berkshire, UK) in 0.1 M phosphate-buffered saline (PBS) at 4°C. The tissue was photographed in wholemount under a fluorescence microscope (Lumar V12 stereomicroscope, Zeiss, Jena, Germany), cryoprotected in 20% sucrose in 0.1 M PBS overnight, embedded in Tissue-Tek OCT compound (Chemi-Teknikk, Oslo, Norway) and frozen in liquid nitrogen. Serial transverse sections (30–50 µm) were made with a cryostat (Leica CM 3050), mounted in 50% glycerol in 0.1 M PBS, and imaged with a laser scanning confocal microscope (Zeiss LSM 5 Pascal) at 543 nm. Stacks of 5–7 confocal images with 5 µm z-axis intervals were generated of each section under a 10x/0.5 objective. Final images were produced by horizontal projection of each stack using LSM Image Browser 4.2 (Zeiss). Labeled neuron profiles (cell somata only) were counted manually in each imaged section and in original z-stacks to corroborate. Profiles were counted in every other section to avoid double-counting neurons intersected by the section.

### Electron Microscopy

Embedding of tissue for electron microscopy was performed as previously described [47]. Briefly, animals were killed and spinal cords dissected out as described above. A 4-segment long piece of the spinal cord centered on the compression site or equivalent segment in sham controls was immediately fixed for 3 hours in 4% PFA, 1% glutaraldehyde (EM grade; TAAB) in PBS, pH 7.4, followed by overnight fixation in 1% PFA, 2.5% glutaraldehyde (EM grade) in PBS, pH 7.4. Vibratome sections of 500 µm thickness were treated with 1% osmium tetroxide (Electron Microscopy Science, Washington, PA, USA) in 0.1 M Na-phosphate buffer, dehydrated in graded ethanols and propylene oxide and embedded in Durcupan ACM (Sigma-Aldrich, St. Louis, MO, USA). Ultrathin sections were cut and contrasted with 10 mg/ml uranyl acetate (Electron Microscopy Science) for 9 min and 3 mg/ml lead citrate for 70 s. The samples were examined with a Tecnai CM10 electron microscope (FEI Company, Hillsboro, OR, USA).

### Electrical Stimulation of Descending Pathways and Dorsal Roots

Fire-polished borosilicate glass (1.2 mm OD, 0.94 mm ID; Harvard Apparatus, Holliston, MA, USA) suction stimulation electrodes with appropriately sized tip diameters were used to deliver short (0.2–0.5 ms) electrical current pulses (40–500 µA) using one of two different stimulators (DS8000, WPI, Sarasota, FL, USA or S48, Grass Instruments, West Warwick, RI, USA). Either single pulses (at least 50 s between two consecutive pulses) or pulse trains (5 Hz for 5 s; at least 3 min between trains) were used for both electrophysiological and optical recording experiments (see below). Vestibulospinal pathways were activated specifically by unilateral stimulation of the VIIIth cranial nerve. General bulbospinal pathways, encompassing the majority of brainstem-spinal cord descending pathways, were activated by unilateral stimulation of the ventral funiculus at cervical level (C)1-C2 or T5 [48], [49]. Lumbar sensory afferents were activated by stimulating a single lumbar dorsal root.

### Electrophysiology

Electrophysiological recording of motoneuron discharges was performed as described previously [49]. Briefly, brainstem-spinal cord preparations were obtained as described above, and fire-polished borosilicate glass suction recording electrodes with appropriately sized tip diameters were placed onto cervical and lumbar ventral roots. Electrical signals were amplified (EXT 10-2F amplifier; npi Electronics, Tamm, Germany), digitized, averaged and analyzed off-line (CED 1401 mkII, SPIKE 2; Cambridge Electronic Design, Cambridge, UK). For recording of compound action potentials, electrode placement was *en passant* a short distance from the root exit site. For recording individual synaptic events, the ventral root potential was recorded by suctioning the cut end of the root entirely into the electrode to make a tight junction with the surface of the spinal cord [49]. Electrophysiological experiments at 8 days after surgery/injury were performed with a reduced *ex vivo* preparation of the isolated spinal cord (from C1 to sacral levels) following the same protocol as above.

### Optical Recording of Synaptically Mediated Calcium Transients

The procedures for optical recording of synaptically mediated calcium transients have been described previously [28]–[31]. Briefly, brainstem-spinal cord preparations were obtained as described above, and spinal motoneurons (MNs) were labeled retrogradely with Calcium Green Dextran Amine (CGDA; Calcium Green-1 dextran, lysine fixable, 3000 MW; Invitrogen) by applying crystals to transected ventral roots and incubating the preparation in oxygenated ACSF at room temperature (24–28°C) for 3 h in the dark. The preparation was then pinned ventral side-up to the Sylgard-coated bottom of a recording chamber (volume 8 ml) and continuously superfused (3–4 ml/min) with oxygenated ACSF at room temperature. Optical recording of calcium transients was performed with an epi-fluorescence microscope (Axioskop; Zeiss) and a high-sensitivity CCD camera (Photometrics Cascade 650; Texas Instruments, Waltham, MA, USA) under a 40x water immersion objective (LUMPlanFl, 0.8 NA; Olympus). For additional equipment details see [29]. Fluorescence intensity was measured within separate regions of interest placed over individual MN somata. Calcium transient waveforms (changes in fluorescence relative to initial fluorescence, Δ*F*/*F*) reflected postsynaptic activity in spinal MNs in response to stimulation of specific inputs, and were generated by averaging 6–10 calcium transients from the same region of interest. The area under the waveform, delimited temporally by the stimulation duration, was measured to give an indication of response magnitude.

### Culture and Viral Transduction of Human Fetal Neural Progenitor Cells (f-NPCs)

F-NPCs (ReNcell VM [50]; Millipore) were cultured on culture dishes coated with laminin (0.1 µg/ml). Lentiviral transduction with an Emerald Green Fluorescent Protein (EmGFP) vector was performed as described previously [51]. A few passages after viral transduction the cells were harvested for transplantation. About 30 min before transplantation, cells were rinsed in PBS and trypsinized to single cell suspension, centrifuged (300 g, 6 min) and resuspended in 1 ml of culture medium, transferred to a 1.5 ml tube and centrifuged a second time (300 g, 6 min) to remove excess medium. As much as possible of the supernatant was removed and the cell pellet was resuspended in the remaining amount and placed on ice until transplantation.

### Fetal Neural Progenitor Cell Transplantation

2 µl of cell suspension was sucked into the tip of a sharp glass micropipette made from glass capillaries (borosilicate 1.2 mm OD, 0.94 mm ID; GC120T-10, Harvard Apparatus) using an electrode puller (Sutter Instruments, Novato, CA, USA). The micropipette was connected to an air-pressure device (PV830 Pneumatic PicoPump, WPI) and mounted on a micromanipulator for placement of the tip into the spinal cord of recently injured SCC animals while still under anesthesia. To eliminate movements of the spinal column caused by respiration, the column was suspended by attaching surgical sutures into the paravertebral muscles caudal and rostral to the laminectomy and hoisting gently from a surrounding frame. A 1.5 µl volume of cells (about 25000 cells) was injected rostral to the compression site at a rate of 1 µl per 5 min to minimize increases in intraspinal pressure. The pipette was then slowly retracted to avoid reflux of the injected cells, and the wound was sutured as described above for spinal cord injury.

### Immunohistochemistry

Immunohistochemistry on 30 µm cryostat sections was performed as previously described [52]. Briefly, sections were blocked for unspecific binding for 1 hour with 10% normal goat serum (NGS) in Tris-buffered saline containing 0.5% (w/v) Triton X-100, pH7.4 (TBST). Sections were further incubated overnight with primary antibodies diluted in 1% NGS in TBST. Anti-neurofilament H (AB1989; Millipore) and Anti-NeuN-biotinylated (MAB377B; Millipore) were used at 1/400 dilution. The sections were rinsed 3x in 1% NGS in TBST and incubated for 1 hour with AlexaFluor-647 goat anti-rabbit (Invitrogen, A21247, diluted 1/400) and streptavidin-CY2 (Jackson ImmunoResearch, 016-220-084, diluted 1/200) in 1% NGS in TBST. The sections were rinsed in 1% NGS in TBST and incubated in PBS containing the nuclear stain Hoechst 33258 (1 µg/ml) for 5 min, further rinsed in PBS and mounted in PBS-glycerol 1∶1.

### Statistics

Statistical comparison of means was performed using the non-parametric Mann-Whitney U test.

## Results

### Creating a Reproducible Procedure for SCC in Neonatal Mouse

In preliminary experiments we determined through exploratory surgery that reproducible dorsoventral compression would be difficult to perform in the neonatal mouse spinal cord without extensive surgery and high mortality. We therefore chose to focus on lateral compression, which could be achieved with less invasive surgery. A drop weight contusion approach as is often used in adult rats [53] was discarded on the basis of size constraints.

In neonatal mice, the thoracic spinal cord is 1.2 to 1.5 mm wide and leaves only a narrow residual intrathecal space to place a compression clip. Thus, we filed down the blades of Yasargil Phynox aneurysm clips to reduce their thickness to 150 µm (Fig. 1A,B). Filing was done manually and therefore the blades could not be assumed to be identical. Moreover, we placed a stopper made from polypropylene tubing on one blade to provide consistent interblade distance on compression. To avoid any asymmetry in the compression of the cord, we performed two compressions switching the orientation of the clip between the compressions. Typically a clip could be used for about 80 compressions (40 animals) before the spring had weakened to the point that the opposing blade no longer met the stopper.

A total of 104 ICR and 19 SCID-ICR mice were used and overall mortality was about 30%, occurring either during surgery (particularly in the early stages of the project, when anesthesia and surgical procedures were being developed, and therefore over-represented in sham controls) or during the first few days thereafter. Fifty-six ICR mice and 19 SCID-ICR mice received SCC (mortality 18/56 and 6/19, respectively), 8 ICR mice received SCT (mortality 2/8), and 40 ICR mice were sham controls (mortality 15/40), which were carried through all surgical procedures except for the SCC itself. Table 1 presents an overview of the various experimental procedures carried out on the surviving animals.

### Tissue Damage Associated with SCC

Immediately following SCC, the compressed region of the spinal cord became darker and swollen, characteristic of hemorrhagic contusion and edema (Fig. 1C). Six to nine hours after the compression, the injured area could be recognized in the dissected spinal cord by the presence of epidural blood forming a dark band (Fig. 1D). At 15–30 days post-injury a pronounced atrophy of the spinal cord was visible at the compression site, and at higher magnification the compression site was translucent whereas normal tissue rostral and caudal to the compression site remained opaque (Fig. 1D). A similar appearance was also observed in the case of full transection injuries (Fig. 1D). In some cases of SCC an endomedullary syringomyelia formed at the injury site (not shown).

**Figure 2:**
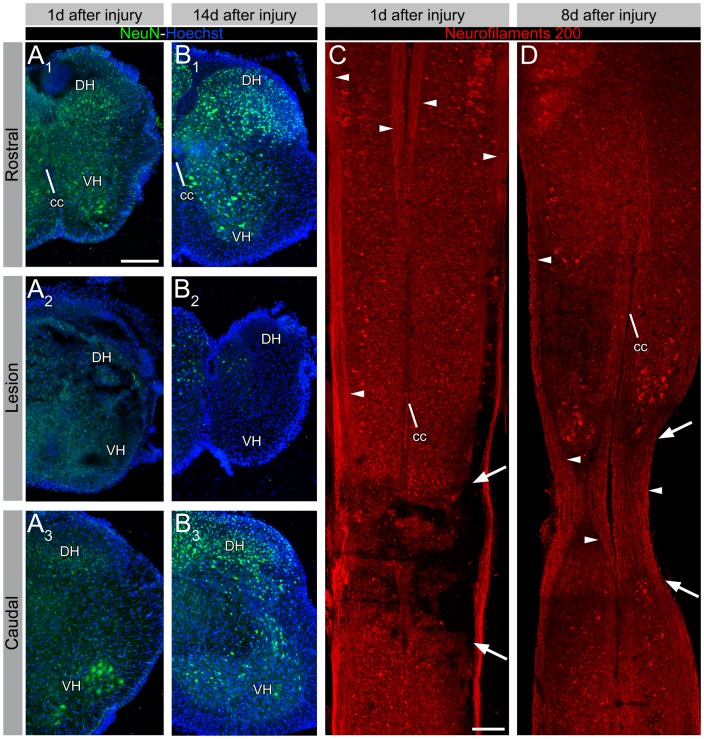
Cellular damage after SCC assessed by immunohistochemistry. (A and B) Immunohistochemistry for NeuN 1 day (A) and 8 days (B) after SCC, seen in transverse sections rostral to, at, and caudal to the lesion. At both timepoints NeuN immunostaining is greatly diminished within the compressed region. (C and D) Immunohistochemistry for 200 kD neurofilaments 1 day (C) and 8 days (D) after SCC, seen in horizontal sections. At 1 day after injury (C) many axons (arrowheads) still traverse the compressed region (arrows), although some fascicles appear to be interrupted. At 8 days after injury (D), there is white matter continuity across the lesion site, with a relatively even density of axons. Abbreviations: cc = central canal, DH = dorsal horn, VH = ventral horn. Scale bars: 200 µm.

To characterize the damage at the cellular level, we used immunohistochemistry to assess the number and distribution of neurons and axons in and near the site of the SCC using antibodies against the neuronal proteins NeuN and 200 kD neurofilament, respectively (Fig. 2). One day after the injury, NeuN immunostaining was diminished within the compressed region relative to areas immediately rostral and caudal (Fig. 2A). By 14 days after injury, well after edema had subsided, NeuN immunostaining was limited to a small fraction of neurons within the compressed region relative to numbers seen more rostral and caudal (Fig. 2B). Neurofilament immunostaining showed that many axons still traversed the compressed region at 1 day after injury, although some axon fascicles appeared interrupted (Fig. 2C). By 8 days after injury, there appeared to be white matter continuity across the compressed region, despite the marked narrowing of the spinal cord (Fig. 2D). Thus, it appeared that most neurons within the compressed region had succumbed to cell death, whereas a substantial proportion of axons was spared.

**Figure 3:**
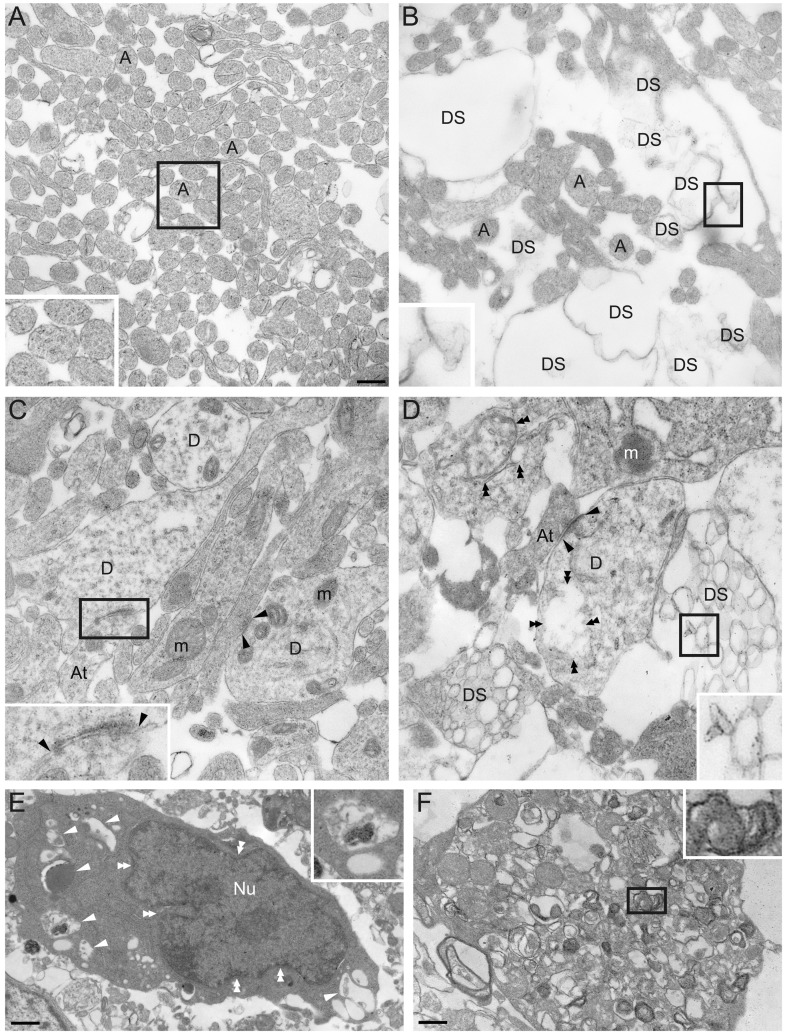
Ultrastructure of SCC and sham control spinal cords 1 day after injury. (A) Axon profiles in the ventrolateral white matter in sham control at P2. Note the presence of filament-like structures within the axon cytoplasm (inset). (B) Ventrolateral white matter in a SCC spinal cord 1 day post-injury. Note the paucity of axons and loosely organized membrane structures (inset). (C) Gray matter in sham control spinal cord at P2. Dendrites, axon terminals, synapses (inset), and subcellular structures such as mitochondria, synaptic vesicles and postsynaptic densities (arrowheads) can be observed. (D) Gray matter in a SCC spinal cord 1 day post-injury. Degenerated structures (inset) are present among less compromised but nevertheless disorganized and discontinuous structures such as dendrites, axon terminals and synapses (double arrowheads). (E) A damaged neuron soma in a SCC spinal cord presents cytoplasmic vacuoles (single arrowheads and inset) and an indented nucleus likely to be in an early stage of nuclear fragmentation (double arrowheads). (F) Cell debris from fragmented plasma membrane and endomembranes. Scale bars: 350 nm (A-D), 760 nm (E) and 390 nm (F). Abbreviations: A = axon; At = axon terminal; D = dendrite; DS = degenerated structure; m = mitochondria; Nu = nucleus.

We also investigated the ultrastructural organization of the spinal cord in and near the compression site with electron microscopy to obtain a general picture of the damage that obtained (Fig. 3). This was performed 1 day after injury to try to capture events occuring during the dynamic phase of tissue damage. In sham controls (n = 2) the ventral and ventrolateral white matter was replete with unmyelinated axons (Fig. 3A). By contrast, in SCC animals (n = 3), the ventral and ventrolateral white matter showed clear signs of axonal degeneration in a substantial proportion of axons (Fig. 3B). In the grey matter of sham controls, structures typical of normal spinal cord tissue at P1 were seen, including axons, axon terminals, dendrites and synapses with an appropriate composition of intracellular organelles and cytoskeleton (Fig. 3C). In the grey matter of SCC animals, by contrast, clear signs of degeneration were present, including structurally disrupted dendrites and axons with poorly organized cytoskeletal elements (Fig. 3D), cell somata with cytoplasmic vacuoles, poorly defined organelles and indented, prefragmentary nuclei (Fig. 3E) and cell debris (Fig. 3F).

**Figure 4:**
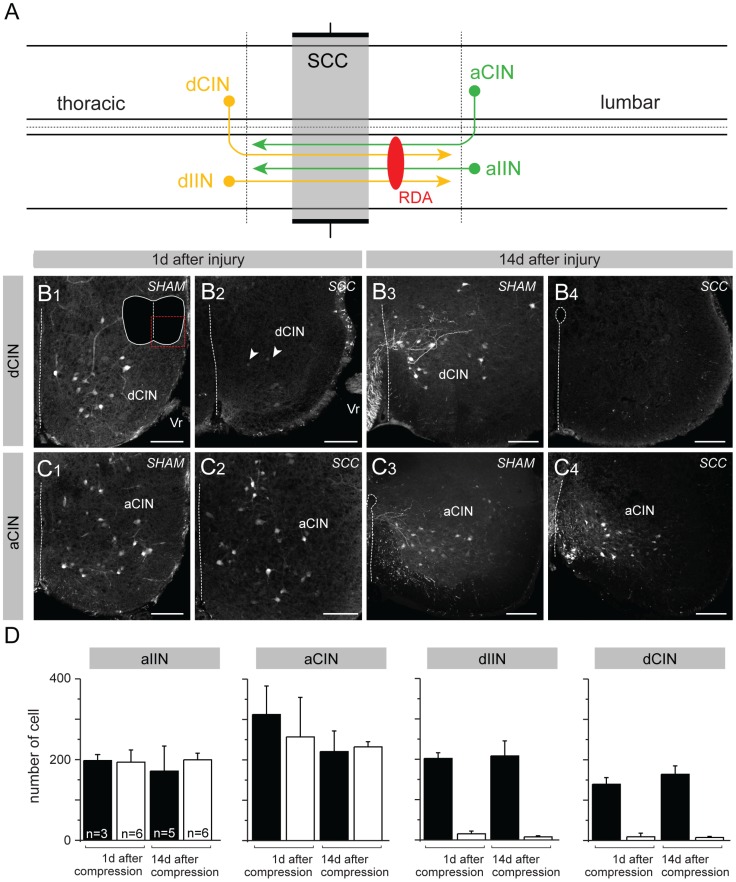
Loss of spinal neurons with descending axons traversing the injury following SCC. (A) Schematic representation of interneurons, propriospinal neurons and projection neurons in the spinal cord based on [34]. The compression was performed in the lower thoracic cord (T10–12, grey area). RDA was applied one segment below the compressed region to label ascending (a) and descending (d) ipsilaterally (I) and contralaterally (C) projecting neurons (INs). (B and C) Representative confocal images of one side of the ventral region of transections containing RDA-labeled dCINs (B) and aCINs (C) in one sham and one SCC mouse 1 day (B1–2, C1–2) and 14 days (B3–4, C3–4) after surgery/injury. Very few are labeled in SCC (arrows, B2) compared to sham controls (B3). The inset in B1 shows the contour of the entire section with the imaged area indicated by the stippled red square. (D) Counts of labeled neuronal profiles of the indicated projection types within a 1 mm stretch of spinal cord immediately rostral to the compressed region (or the equivalent in sham animals) and immediately caudal to the RDA application site (see also Fig. S1). Bars represent averages of total counts taken from every other section in the relevant 1 mm length of spinal cord, with number of animals indicated in the first graph. Error bars represent standard deviations. Differences in counts of dIINs and dCINs between sham control and SCC animals are significant at p<0.025 at 1 day after surgery/injury and at p<0.01 at 14 days after surgery/injury (Mann-Whitney U-test, U = 0 in both cases). Scale bars: 100 µm. Additional abbreviations: Vr = ventral root.

To assess damage to identifiable populations of spinal neurons, we applied the retrograde axonal tracer RDA [45], [46] unilaterally just caudal to the compressed region 1 day or 14 days after SCC or sham surgery. This labels interneurons, propriospinal neurons and projection neurons, which for simplicity we will refer to collectively as interneurons (INs; see [34] for a general discussion of this terminology). We quantified the number of ipsilaterally projecting and commissural INs with descending axons (dIINs and dCINs, located rostral to the application site) or ascending axons (aIINs and aCINs, located caudal to the application site, Fig. 4), as previously described in the neonatal mouse and rat [34]. The expectation was that specifically the dIINs and dCINs should be diminished in number because their axons were damaged at a site between their somata and the RDA application site (Fig. 4A). Figures 4B and 4C show representative retrograde labeling of dCIN and aCIN populations in sham and SCC animals, illustrating the dramatic decrease in labeled dCINs at both post-injury timepoints. Retrogradely labeled neurons were then counted in the region caudal to the RDA application site (aIINs and aCINs) and rostral to the compressed region (dIINs and dCINs; these were only counted rostral to the compressed region, not within the compressed region, for appropriate comparison to sham controls, see Fig. S1). In sham controls we found a normal distribution of each interneuron type, with numbers decreasing gradually with distance from the application site (n = 3, see Fig. S1). In SCC mice (n = 6), aIINs and aCINs exhibited the same distribution as in sham controls, decreasing in number with distance caudal to the application site. By contrast, dIINs and dCINs were virtually absent within the compressed region (as expected, since we had already seen a dramatic loss of neurons within this region, Fig. 2B), and dramatically diminished rostral to the compressed region compared to sham controls (Fig. 4D).

### Behavioral Assessment Shows an Initial Phase of Hindlimb Paralysis Followed by a Gradual, Substantial Recovery

**Figure 5:**
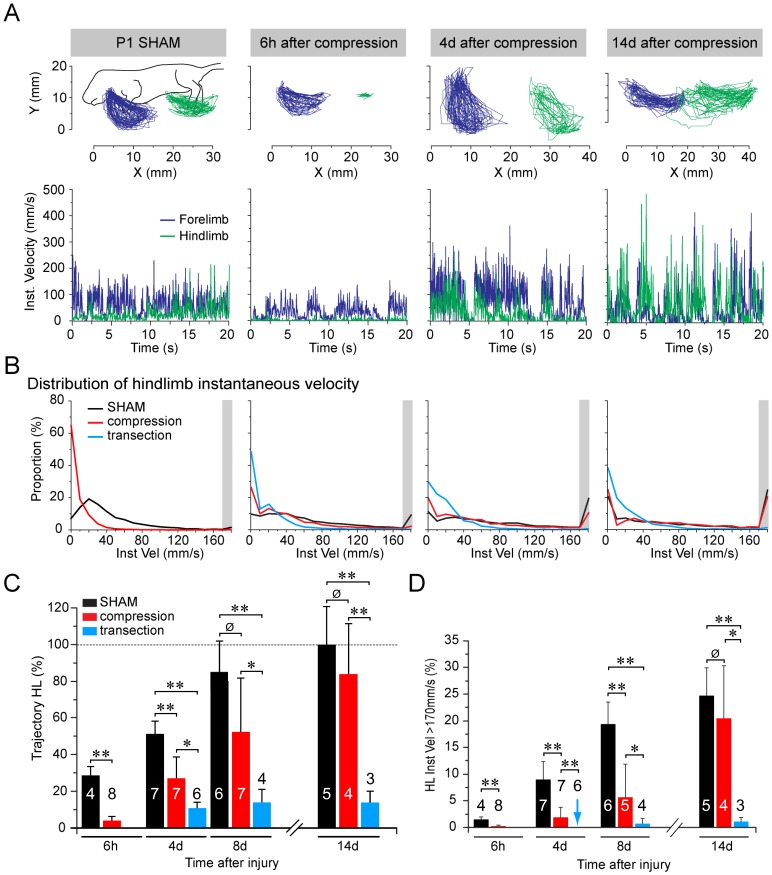
Recovery of hindlimb motility following spinal cord injury. (A) Kinematic assessment of trajectories of forepaws (blue traces) and hindpaws (green traces) during air stepping test of sham control and SCC mice at 3 times post-injury (post-surgery for sham controls). Upper panels show actual trajectories, and lower panels show instantaneous velocities. (B) Distribution of hindlimb instantaneous velocities in sham control (black), SCC (red) and SCT (blue) mice during 20 s video sequences, expressed as the proportion of trajectories with different velocities. Grey zone at far right of each graph indicates the bin containing instantaneous velocities greater then 170 mm/sec. This bin was distinct in that the proportion of velocities over this value increased markedly both in sham control and SCC mice over time. (C) Mean hindlimb trajectory amplitudes in sham control, SCC and SCT mice at different post-injury times normalized to sham controls at 14 days post-surgery. Numbers in or over bars represent numbers of animals tested. Error bars represent standard deviations. Statistical significance of differences of means tested by Mann-Whitney U-test: p≤0.002 (***), p = 0.01 (**), p = 0.09 (Ø). Note that p value for difference between sham control and SCC mice reaches non-significance by 8 days post-surgery. (D) Proportions of instantaneous velocities >170 mm/s in sham control, SCC and SCT mice at different times post-surgery/injury. Blue asterisk represents a measurement of value zero, otherwise the lack of a bar indicates that no measurement was made (which was the case for 6 h post-injury for SCT mice). Numbers in or over bars represent numbers of animals tested. Error bars represent standard deviations. Statistical significance of differences of means tested by Mann-Whitney U-test: p≤0.003 (***), p≤0.025 (**), p>0.1 (Ø). Note that p value for difference between sham control and SCC mice reaches non-significance by 14 days post-surgery.

Since early postnatal mice cannot perform weight-bearing locomotion, we tested the effect of SCC on motor behavior using an air-stepping assay (modified from [42]) in which mice were suspended in a soft harness and stimulated by touching the nose. Movements of all 4 limbs were video recorded at 25 Hz and analyzed kinematically. Within 6 hours after SCC there was a dramatic deficit in hindlimb motility, measured as movement trajectory and instantaneous velocity (Fig. 5A, n = 8), compared to sham controls. We then compared behavioral recovery at different time points in SCC mice (4 days and 8 days after SCC, n = 7 each; 14 days after SCC, n = 4), in SCT mice (4 days after SCT, n = 6; 8 days and 14 days after SCT, n = 3 each), and in sham controls (6 hours after surgery, n = 4; 4 days after surgery, n = 7; 8 days after surgery, n = 6; 14 days after surgery, n = 5). Reflecting the ongoing motor development during the postnatal period, in the sham control mice there was an increase in trajectory amplitude and the proportion of rapid movements (>170 mm/sec instantaneous velocity) during the 2 weeks following surgery (Fig. 5B, C; Fig. S2A, B). SCT mice showed a dramatic loss of hindlimb motility 6 hours after the injury similar to that in SCC mice (Fig. 5A, C). From this deficit, SCC mice exhibited a marked recovery of rapid movements (>170 mm/sec) and trajectory amplitudes (Fig. 5C, D). Average trajectory amplitudes and proportions of movements exceeding 170 mm/sec had reached 80% of the levels exhibited by sham controls by 14 days post-injury (Fig. 5C, D). By contrast, SCT mice exhibited some recovery of trajectory amplitude but no recovery of rapid movements by 4 days post-injury, and exhibited little if any additional recovery thereafter (Fig. 5 C, D).

To assess behavioral recovery at later stages, we videotaped sham and SCC animals during a single-track locomotion test 24 days after surgery/injury. At this time, animals in the 2 groups were virtually indistinguishable when performing this test, and an analysis of gait showed no obvious differences in the stance durations of the forepaws or the hindpaws (Fig. S2C, D).

### Electrophysiological Assessment Shows an Initial Loss of Bulbospinal Inputs to Lumbar Motoneurons Followed by Recovery

One of the advantages of neonatal mice is the possibility to make *ex vivo* preparations of the isolated brain stem and spinal cord in which descending motor pathways can be assessed physiologically [28]–[32], [49]. As a first step towards assessing the temporal pattern of recovery in general bulbospinal inputs to lumbar motoneurons (MNs), we isolated the brain stem and spinal cord from SCC and sham control mice and stimulated electrically the ventral funiculus (VF) at C1 while recording extracellularly from lumbar ventral roots *en passant* (Fig. 4A [48], [49]). Recordings from cervical ventral roots were included to compare with responses to bulbospinal inputs that had not been damaged by the SCC.

**Figure 6:**
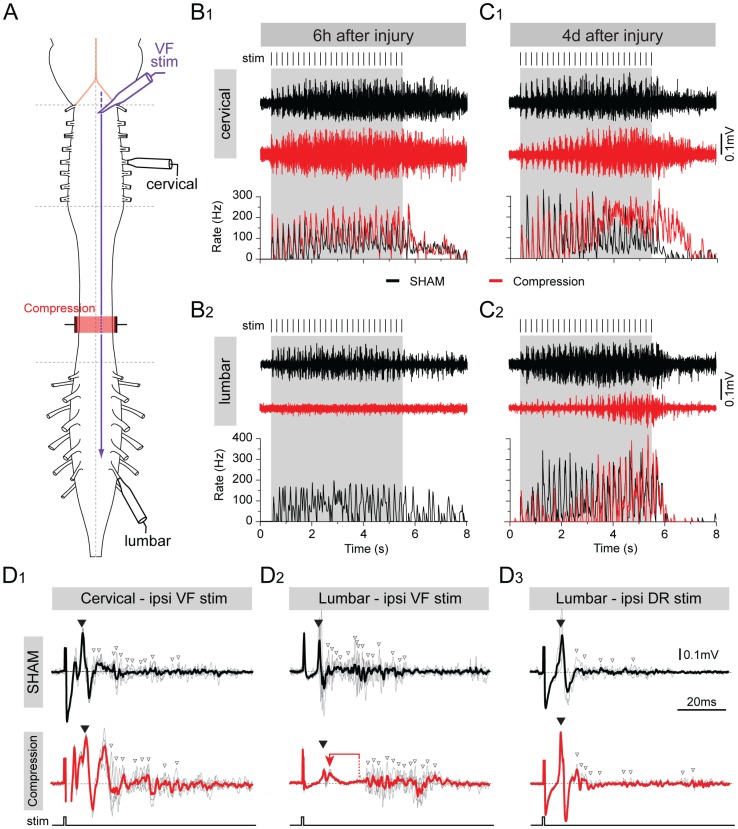
Recovery of responses to general bulbospinal pathway stimulation following SCC, assessed with electrophysiology. (A) Schematic of the *ex vivo* preparation of the brain stem and spinal cord showing the site of bulbospinal pathway stimulation in the ventral funiculus (VFstim), the compressed region (red zone) and the recording suction electrodes placed on cervical (above the injury) and lumbar (below the injury) ventral roots. (B and C) Ventral root responses during stimulus trains (grey box, 25 pulses over 5 s, individual stimulus pulses indicated above) in cervical (B1, C1) and lumbar (B2, C2) ventral roots in 6 h post-surgery sham control (B, black traces), 6 h post-injury SCC (B, red traces), 4 day post-injury sham control (C, black traces), and 4 day post-injury SCC mice (C, red traces). Upper traces show raw recordings, graphs show instantaneous spike firing rates. (D) Tight seal recordings from cervical (D1) and lumbar (D2,3) ventral roots following single pulse stimulation of the ventrolateral funiculus (D1, D2) or the lumbar dorsal root (D3) in sham control (black traces) and SCC mice 4 days after surgery/injury. Filled black arrowhead indicates the monosynaptic component of the response, open arrowheads indicate polysynaptic components. The red arrow in D2 shows the increased delay between monosynaptic and first polysynaptic component 4 days post-injury.

In sham controls (n = 3), repetitive stimulation (25 pulses in 5 s) elicited a repetitive discharge in cervical roots (Fig. 6B1, C1, black traces) and lumbar roots (Fig. 6B2, C2, black traces), starting with the first and ending a few seconds after the last stimulation pulse. The compound action potential discharge rate was generally similar in cervical and lumbar roots (≈200 Hz) with no observable difference between 6 hours (Fig. 6B1, C1) and 4 days (Fig. 6B2, C2) post-surgery.

By contrast, in SCC mice, VF stimulation at C1 6 hours after injury (n = 5), still elicited a normal discharge in cervical roots (Fig. 6B1, red trace), but no discharge in lumbar roots (Fig. 6B2, red trace). By 4 days post-injury (n = 4), the lumbar root discharge had partially recovered (Fig. 6C2, red trace), but was delayed and less intense than in sham controls (Fig. 6C2, black trace).

To characterize the responses in more detail, we made recordings with the electrodes tightly sealed to the ventral root exit point and used single pulse stimulation. This recording configuration, due to proximity of the electrode to the MN somata, provides information about synaptic responses in the MNs. Responses to VF and dorsal root stimulation were compared to identify mono- and polysynaptic components ([54]; Fig. 6D). In both cases, an initial large amplitude waveform could be identified as the monosynaptic component (Fig. 6D, black arrowhead), followed by a series of smaller waveforms constituting polysynaptic components (Fig. 6D, white arrowheads). Monosynaptic components to VF stimulation had consistently shorter latencies in cervical than lumbar roots, and increased in amplitude with increasing stimulation intensity, with no change in latency (not shown; see [54]). Polysynaptic components were more variable in duration but did not vary noticeably in amplitude with stimulation intensity (not shown).

Four days after injury/surgery, the cervical monosynaptic component in response to VF stimulation was similar in SCC (Fig. 6D1, black trace) and sham control mice (Fig. 6D1, red trace). By contrast, the lumbar monosynaptic component in response to VF stimulation had a substantially lower magnitude in SCC (Fig. 6D2, black trace) than in sham control mice (Fig. 6D2, red trace). Moreover, an abnormally long delay was often seen between the monosynaptic and the polysynaptic components in the SCC mice (Fig. 6D2, see red arrow). Monosynaptic and polysynaptic responses following same-segment dorsal root stimulation were not noticeably different in SCC and sham mice (Fig. 6D3 [54]), indicating that only the descending inputs had been affected by the injury. Thus, when electrophysiological responses reappear during the first 4 days of recovery from SCC they exhibit an altered pattern of monosynaptic and polysynaptic components.

Eight days after surgery, responses to VF stimulation had become much more similar in SCC and sham control mice, both in terms of overall discharge during train stimulation, the amplitudes of monosymaptic inputs and the latencies of polysynaptic inputs (Fig. S3). Thus, in parallel with behavioral recovery, there is a recovery of the strength of responses of lumbar MNs to general bulbospinal inputs.

### Optical Recording Demonstrates Post-lesional Plasticity in Descending Inputs to Lumbar MNs

**Figure 7:**
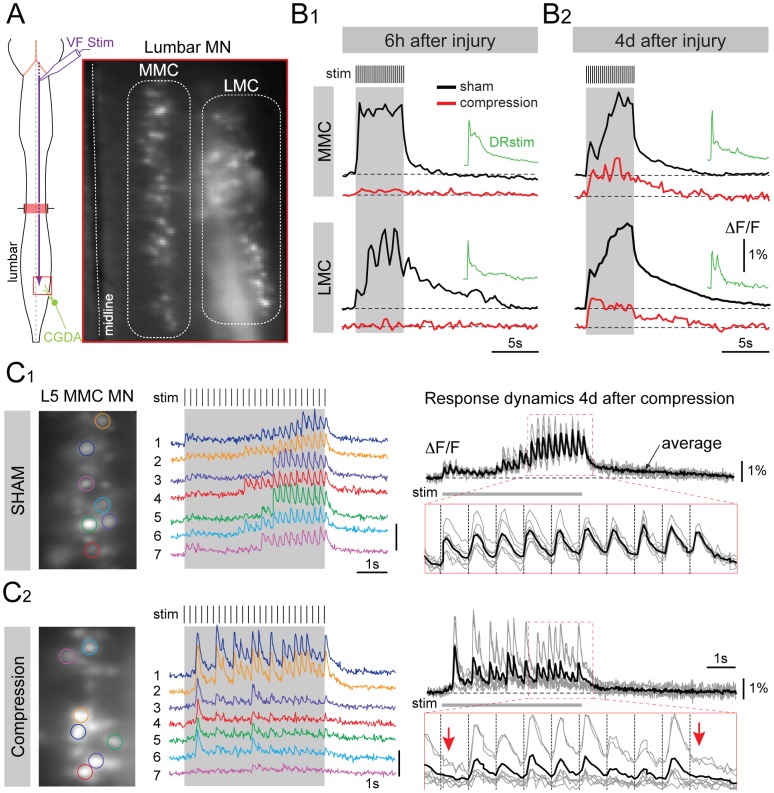
Recovery of responses to general bulbospinal pathway stimulation following SCC, assessed with optical recording. (A) Schematic of the *ex vivo* preparation of the brain stem and spinal cord showing the site of bulbospinal pathway stimulation in the ventral funiculus (VF Stim) and the CGDA labeling of the L5 ventral root, and a photograph of the CGDA labeled lumbar MNs in the medial motor column (MMC) and lateral motor column (LMC). (B) Low temporal resolution (4 Hz frame rate) optical recordings of calcium transients elicited in MMC and LMC MNs in response to VF stimulation (same parameters as in Fig. 6) 6 h and 4 days after surgery/injury in sham control mice (B1 and B2, black traces) and SCC mice (B1 and B2, red traces). Green traces show responses to stimulation of the L5 dorsal root in SCC. ΔF/F represents fluorescence change in % relative to baseline. (C) High temporal resolution (50 Hz frame rate) recordings from individual L5 MMC MNs in response to VF stimulation in sham control (C1) and SCC (C2) mice 4 days after surgery/injury. Each colored trace shows the calcium transients in one of the individual MNs outlined by a colored region of interest in the photographic images to the left. Black and grey traces (right column) represent the superimposed individual traces (grey) and the averaged response (black) from the same MNs, with the expanded traces (outlined in red) showing the responses to the last 10 pulses of the train. Red arrows in the expanded trace of C2 (4 days post-injury) indicate absent calcium transients in response to the 15th and the last pulses of the train.

**Figure 8:**
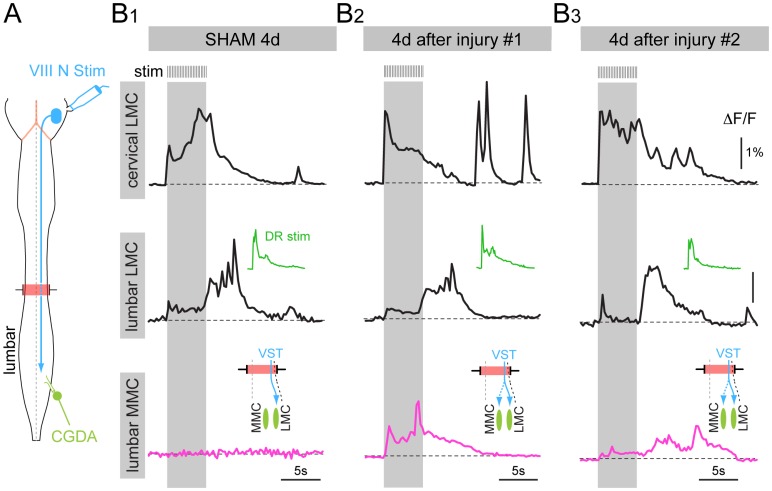
Reorganization of vestibulospinal connections to lumbar medial and lateral motor columns following SCC. (A) Schematic of the *ex vivo* preparation of the brain stem and spinal cord showing the site of vestibular nerve stimulation (VIII N Stim) and the CGDA labeling of the L5 ventral root. (B) Low temporal resolution (4 Hz frame rate) optical recordings of calcium transients elicited in LMC MNs (black traces) and MMC MNs (red traces) in response to train stimulation of the vestibular nerve (same parameters as in Fig. 6) in one sham control mouse (B1) and two SCC mice (B2, B3) 4 days after surgery/injury. ΔF/F represents fluorescence change in % relative to baseline. The small green traces (DR stim) show responses to stimulation of the L5 dorsal root. Insets above the MMC traces show the apparent reorganized synaptic connectivity from lateral vestibulospinal tract (VST) to LMC and MMC.

We have previously shown that optical recording of synaptically mediated calcium transients facilitates a high-throughput assessment of the spatial pattern of connections from the reticulospinal tract and the vestibulospinal tract onto spinal MNs [28], [30]–[32]. Here, we used the same approach to study the functional recovery of general bulbospinal inputs (Fig. 7) and vestibulospinal inputs (Fig. 8) onto lumbar MNs following SCC.

Stimulating the VF in sham control mice activated MNs in both the lateral motor column (LMC) and medial motor column (MMC) of lumbar segments (Fig. 7B1, B2, black traces), as expected from our earlier work showing that medullary and pontine reticulospinal inputs target both motor columns in the lumbar cord [28], [55]. By contrast, 6 hours after SCC, responses in both motor columns were completely abolished (Fig. 7B1, red trace). By 4 days post-injury, responses in both motor columns had reappeared, but were weaker (Fig. 7B2, red trace) and more fatigable to repetitive stimulation (not shown) than in sham controls (Fig. 7B2, black trace). Responses in cervical MNs were not affected by SCC (not shown), nor were responses in lumbar MNs to same-segment dorsal root stimulation (Fig. 7B1, B2, green traces), indicating that the effect of SCC was specifically on the bulbospinal projections to the lumbar cord.

As a means to assess the potential reorganization of descending inputs following SCC, we also examined response heterogeneity by comparing responses to repetitive stimulation in individual MNs recorded at higher temporal resolution (50 Hz, Fig. 7C1 and C2). In sham control mice, responses were very similar in different MNs, as shown by the similarity of the different color-coded traces in Fig. 7C1 (middle panel) and the low variability of individual responses (grey traces) around the mean response (black trace) in Fig. 7C1 (right panel). By contrast, 4 days post-injury, responses in individual MNs were substantially more variable, including response failures (Fig. 7C2), which were rare in sham control mice. Thus, during recovery from SCC there was much more heterogeneity in the bulbospinal inputs to individual MNs, indicating a potential reorganization of synaptic connections.

To further examine potential reorganization of descending inputs following SCC, we investigated the pattern of vestibulospinal inputs onto MNs in the lumbar LMC and MMC (Fig. 8). Our earlier work had shown that, in contrast to reticulospinal inputs, the lateral vestibulospinal tract selectively activates LMC MNs in ipsilateral lumbar segments, with no detectable calcium transients in ipsilateral MMC MNs [30]. As expected, on the ipsilateral side of the spinal cord in sham control mice there was a selective activation of the lumbar LMC, with no detectable response in the MMC (Fig. 8B1). As was the case for general bulbospinal inputs, responses in the LMC to vestibulospinal inputs were completely eliminated 6 hours post-injury (not shown). By contrast, by 4 days post-injury responses in the LMC had returned, but were also now detected in the MMC (Fig. 8B2, B3). This aberrant connection provides clear evidence for a reorganization of synaptic connections following SCC.

### Human Fetal Neural Progenitor Cells Transplanted into the Compression-injured Spinal Cord Can Survive and Begin to Differentiate

**Figure 9:**
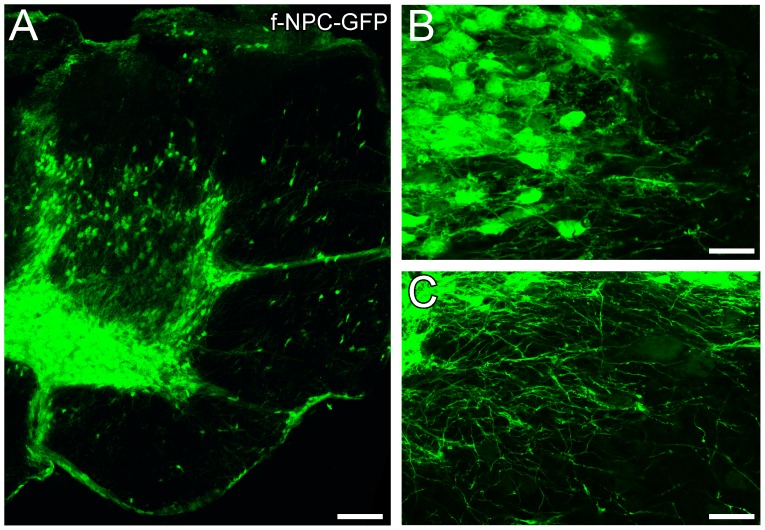
Integration of human fetal neural progenitor cells following transplantation into neonatal spinal cords with SCC injuries. (A) Transverse section showing GFP-positive f-NPC-derived cells 29 days post-injury. (B, C) Higher magnification views of GFP-positive f-NPC-derived cells showing morphological differentiation including the extension of thin, ramified processes. Scale bars: 20 µm (A), 32.5 µm (B, C).

Since one of our motivations to develop the neonatal SCC model is to establish a platform for testing the capacity of human stem and progenitor cell-derived neurons to integrate functionally into spinal neural circuits, as a proof of principle we asked whether human fetal neural progenitor cells (f-NPCs) can be successfully transplanted into the spinal cord of SCC mice and survive and differentiate there. In this experiment we transplanted undifferentiated GFP-positive human f-NPCs near the compressed region a few minutes after SCC (n = 5). Twenty-nine days later we dissected out, fixed and sectioned the spinal cord and found by laser scanning microscopy many GFP-positive cells in the region near the injury site, both in the grey matter and the white matter (Fig. 9A). Because they expressed GFP, it was possible to see that the cells had differentiated morphologically and that some had processes that extended up to several hundred microns from the somata (Fig. 9B, C). Thus, the neonatal mouse SCC model clearly supports the survival and differentiation of human progenitor cells. This provides an opportunity in future experiments to characterize the formation of functional synapses onto these neurons by different presynaptic sources including descending projections from the brain stem.

## Discussion

### The Rationale for using a Neonatal SCI Model to Study Post-injury Plasticity

Brain plasticity represents a continuum of processes that spans from the formation of synapses and networks in prenatal and early postnatal life to their ongoing modification during adult life. Although the capacity for plasticity may vary, a number of underlying mechanisms appear to be relevant throughout the lifespan, such as the ability of axons to sprout new collateral branches, the modulation of neurite growth and synaptogenesis by neurotransmitters and growth factors, and the regulation of synaptic strengths by patterned activity. One of our working hypotheses in developing a neonatal SCI model is that the envelope of plasticity exhibited by the neonatal spinal cord encompasses the plasticity of which the adult spinal cord is capable. Indeed, it might be argued that a strong strategy for promoting regeneration in the adult spinal cord is to reinstate an earlier developmental condition, and many current approaches, such as knocking down axon growth inhibitors in myelin, upregulating trophic factor expression, and introducing stem or progenitor cells [41], represent precisely that. Describing the scope of post-injury plasticity in the neonatal spinal cord is in this sense equivalent to mapping the possibilities that might be made available experimentally and clinically to the adult spinal cord.

Of more direct relevance is the existence of pediatric spinal cord injuries that are less well characterized than adult injuries both in terms of etiology, pathological development and functional sequelae. There is a general lack of experimental models for pediatric spinal cord injuries. The model presented here provides a platform that more likely reflects the pathogenetic and recovery processes characteristic of the developing human than do adult animal SCI models.

One of the principal advantages of establishing a model in the neonatal mouse spinal cord is the opportunity to utilize certain methodologies that are much more difficult to apply to the adult spinal cord. Because of its small size, it is very easy to make *ex vivo* preparations of the neonatal spinal cord that can be experimentally manipulated with great precision, for example in the tracing of neuronal projections or the placement of electrodes for stimulation and recording [34], [56]. A valuable approach that we have helped develop recently is the optical recording of neuronal activity. This facilitates a high-throughput analysis of synaptic connections, for example between bulbospinal axons and target spinal neurons [29], [35]. Using optical recording it is possible to map out, relatively quickly, entire systems of connections in the neonatal spinal cord that take much longer to elucidate using more conventional electrophysiological techniques (see for example [30]). It is important to point out in this respect that, although new approaches are being developed to facilitate optical recording also in the adult spinal cord [57], there are significant technical hurdles to implementing these in the intact adult spinal cord which make the neonatal spinal cord a more attractive experimental system. These include the degree of myelination and the sheer volume of the tissue in the adult cord, both if which severely hamper optical recording, as well as the difficulty in maintaining *ex vivo* preparations of the whole adult cord. Thus, despite the obvious fact that the neonatal and adult spinal cord differ in many respects, studying brainstem-spinal cord circuits and their post-injury plasticity in the neonatal mouse will provide, rather quickly, a wealth of information that should increase insight into the synaptic organization and potential for reorganization of the adult spinal cord.

The main aims of this initial study have thus been to establish a model for studying pediatric spinal cord injuries, as well as to demonstrate the advantages of using the neonatal mouse spinal cord for integrated studies of SCI, including high-throughput assessment of synaptic connectivity. We intend to use this model both in the context of post-SCI plasticity and in the context of integrating stem and progenitor cell-derived progeny into neuronal circuits.

To achieve these aims, we chose to make a compression injury of the spinal cord. This kind of injury is known to spare a certain proportion of axons and would thus provide a situation favorable for axon sprouting and the remodeling of connections. Sprouting of spared axons and/or remodeling of synaptic connections without the regrowth of long descending axons have been described in adult rats and cats with incomplete spinal cord lesions [1]–[8], [16] and in neonatal rats with complete spinal cord transections [19], [58]. Although there are exceptions, in most cases reorganization of spinal circuitry has been deduced from behavioral and anatomical signs, rather than explained in terms of functional synaptic connections between specific axons and specific target spinal neurons. This is where we believe the neonatal mouse model can provide more comprehensive information.

### Evidence for Reorganization in the Context of the Developing Spinal Cord

The SCC mice exhibited substantial functional recovery during the initial two weeks after injury that involved a nearly complete resolution of hindlimb paralysis and an acquisition of weight-bearing locomotion. This was paralleled by the reappearance of mono- and polysynaptic connections from general bulbospinal axons and, more specifically, from lateral vestibulospinal tract axons, onto lumbar MNs. The rate at which this recovery appeared was faster than the ongoing developmental improvement seen in sham control mice, so it is not merely a reflection of continued development. Moreover, although we cannot rule out a contribution to the recovery by the later arrival of descending axons that have not reached thoracic levels at the time of the injury (corticospinal axons are not fully represented at lumbar levels until about P14, for example [59]), the fact that mice with a full SCT recovered hardly at all by comparison (tissue continuity is regained after SCT) suggests that continued development alone cannot account for all of the recovery. Rather, it would appear that at least some of the recovery must be ascribed to the axons that were spared in the SCC situation. These would have the opportunity to sprout new collaterals, innervate novel targets including propriospinal neurons [33], and thus give rise to novel circuits.

Direct evidence for the formation of novel connections following SCC is the appearance of vestibulospinal responses in MNs of the ipsilateral MMC. This connection has not been seen in previous studies of vestibulospinal inputs to lumbar MNs in uninjured mice of the same age and strain [30], nor was it ever seen in sham controls here. The functional significance of this aberrant connection is unclear, but since the MMC innervates trunk musculature, it might lead to the activation of proximal hip-stabilizing muscles. This could conceivably confer an advantage for generating locomotor movements in a situation where some of the original descending connections to MNs innervating more distal musculature have been lost. It will be important in future experiments to follow this and other potential examples of synaptic reorganization to later stages to determine whether they are stable or subject to ongoing modification.

As is characteristic for many regions of the developing CNS [60], the degree of plasticity seen here is likely to diminish as the spinal cord matures. Several developmental watersheds occuring in the spinal cord during the first one to two weeks of postnatal life will contribute to limiting plasticity. Myelination accelerates towards the end of the first postnatal week [61]–[63], and this will limit axon growth and sprouting [40], [64]. Perineuronal nets also appear in the second postnatal week [65] and would be expected to limit the capacity for synaptic remodeling. It will therefore be important to chart the degree of plasticity in specific circuits as the spinal cord matures to determine whether these restrictive events exert homogeneous effects on all spinal neurons or whether some circuits or neuron types maintain a more privileged state. More information about how and where plasticity is downregulated would contribute to defining strategies for reintroducing developmental levels of plasticity in the adult spinal cord [16] in a targeted fashion so as to avoid maladaptive plasticity [13].

### Technical Considerations Regarding Compression Injury in Neonatal Mice

The original clip-compression model described in adult rats used a modified Kerr-Lougheed aneurysm clip [66]–[68]. An advantage of this clip, as well as of drop-weight contusion apparatuses [53], is that they generate dorsoventral compression, which is more relevant in a clinical context than lateral compression, at least for injuries in adults. However, generating SCC in the neonatal mouse involves small structures, narrow anatomical spaces, and fragile patients, and it was necessary to fashion new tools for the purpose. In preliminary experiments, we found that gaining sufficient access to the vertebral column to generate dorsoventral compressions required more extensive laminectomies, which led to higher morbidity, and lifting of the cord within the vertebral column, which led to less controlled compressions. Thus, we decided to use lateral compression instead, which we could perform with less invasive and shorter duration surgery. To create an appropriate tool for the compression, we trimmed the blades of a Yasargil aneurysm mini-clip down to 150 µm thickness so that they could be inserted on either side of the spinal cord within the vertebral column. Our EM and retrograde labeling results indicate that the injury we obtained is symmetrical, affects white matter around the entire perimeter of the cord, and in particular damages descending and propriospinal axons in the ventral, ventrolateral and lateral funiculi.

Another advantage of the modified Kerr-Lougheed aneurysm clip developed by the Tator group is the stability of its closing strength over the course of many compressions. In addition, the closing strength can be adjusted by changing the position of the spring or by using springs of different tensions [68]. Although we did not measure compression strength directly, we standardized the blade separation of our clip and therefore the severity of the compression by placing a stopper on one of the blades. In principle, different stoppers can be used to change the blade separation and thus the severity of the compression. However, more severe compressions can also be performed with the same stopper by incrementing the number of compressions or increasing the compression time. An important aspect of clip compression approaches is that compression strength may decrease with use as the clip spring weakens, which affects both compression force and distance. Our clip could be used for as many as 80 compressions before the spring weakened to the point that the stopper was no longer reached.

### Stem and Progenitor Cell Transplantation and Potential Integration into Functional Circuits

In the context of spinal cord injury and disease, the two main potential applications of stem and progenitor cells are the creation of more favorable environments for repair (promoting axon growth and sprouting, stimulating endogenous stem cells, alleviating inflammatory events) and the replacement of neurons or glial cells that have been destroyed [41], [69]–[71]. Our focus is on the replacement of neurons, and the motivation for this interest is that some spinal cord injuries and diseases may destroy specific neuron populations or circuits whose functions may not be recoverable through adaptive plasticity alone. Our results here underscore this notion, since we find a substantial, protracted loss of neurons within the injured region. Numerous studies have shown that transplanted stem or progenitor cells can give rise to differentiated neuronal progeny in the injured spinal cord (see for example [72] and references therein), but relatively little information is available about the extent to which these exogenous neurons integrate into functional circuits, and in particular whether they receive supraspinal inputs or transmit signals across a lesion. In the neonatal mouse SCC model this can be accomplished rapidly through optical recording of exogenous neurons that express genetically encoded activity probes such as the GCaMPs [73].

### Future Experiments

One of the versatile features of this SCC model is that the optical recording approach can be applied to specific subpopulations of spinal neurons at single cell resolution. Here we have characterized responses in individual motoneurons of the two different motoneuron columns, the MMC and LMC, at different levels of the spinal cord. The same approach can be used to record from local spinal interneurons [29]–[31], which are known to play pivotal roles in recovery of function after incomplete SCI in the adult spinal cord (reviewed in [33]). This can be done using conventional calcium probes as used here, or using genetic calcium probes to restrict recording to specific genetically defined interneuron subpopulations. Our next goal is to take advantage of this unique opportunity to characterize the post-injury reorganization of spinal circuitry at the level of identified subsets of interneurons.

## Supporting Information

Figure S1 **Distribution of retrogradely labeled spinal neurons in a single sham (black dots) and a single SCC (white dots) spinal cord 1 day after surgery/injury.** Each dot represents the number of RDA-labeled neuronal profiles in a single section taken at the indicated level along the length of the spinal cord. The grey area represents the compressed region, and the red area the RDA application site. The two sets of sections were aligned using the rostral terminus of the RDA application site as zero. Note that in the SCC mouse neurons are virtually absent within the compressed region, and that there are few labeled neurons with axons descending through the compressed region to the RDA application site.
(TIF)

Figure S2 **Developmental profile of air stepping in sham control mice, and gait analysis of sham and SCC mice.** (A) Kinematic assessment of the trajectories (A) and instantaneous velocities (B) of forepaws (blue traces) and hindpaws (green traces) during air stepping by sham mice at 3 times after surgery. (C and D) Gait analyses comparing sham and SCC mice 24 days after surgery/injury. (C) Representative stance durations of the 4 paws of one SCC mouse (red) and one sham control mouse (black) during voluntary walking along a track. (D) Average stance durations of the 4 paws of SCC and sham mice during single track locomotion.
(TIF)

Figure S3 **Electrophysiological activity in descending motor pathways 8 days after surgery/injury.** (A) Activity pattern in lumbar ventral roots in response to electrical ventral funiculus (VF) train stimulation (25 pulses during 5 s, pulse duration 0.2 ms, x2T–x3T, where T = threshold for generating a response) in one sham control mouse (top black trace) and one SCC mouse (bottom red trace). Bottom plots in A show the instantaneous firing frequency. (B) Activity pattern in lumbar ventral roots in response to electrical single pulse stimulation of the ipsilateral ventral funiculus (ipsi VF, top pair of traces) and ipsilateral dorsal root in the same segment (ipsi DR, bottom pair of traces) in one sham control mouse (black traces) and one SCC mouse (red traces).
(TIF)
