# Supplementary figures and images for: A Neonatal Mouse Spinal Cord Injury Model for Assessing Post-Injury Adaptive Plasticity and Human Stem Cell Integration
Source: PLoS One. 2013 Aug 19;8(8):e71701. doi: 10.1371/journal.pone.0071701 (PMC3747194; doi:10.1371/journal.pone.0071701)

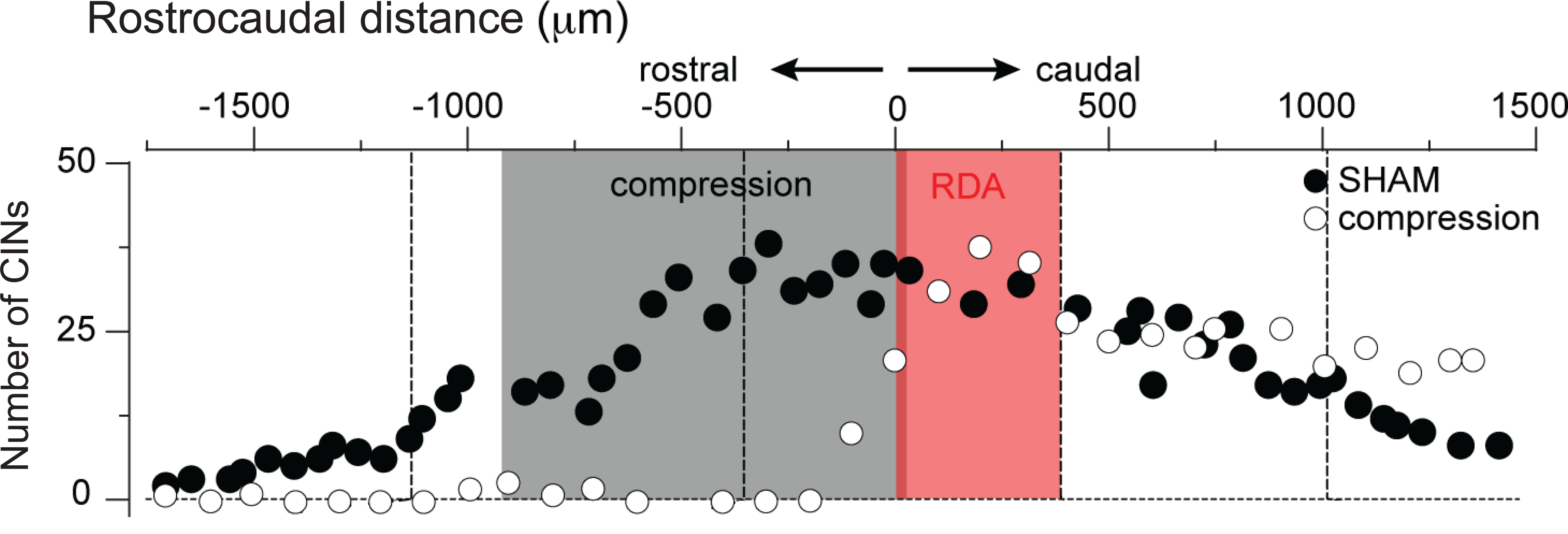

Supplement: Figure S1 — Distribution of retrogradely labeled spinal neurons in a single sham (black dots) and a single SCC (white dots) spinal cord 1 day after surgery/injury. Each dot represents the number of RDA-labeled neuronal profiles in a single section taken at the indicated level along the length of the spinal cord. The grey area represents the compressed region, and the red area the RDA application site. The two sets of sections were aligned using the rostral terminus of the RDA application site as zero. Note that in the SCC mouse neurons are virtually absent within the compressed region, and that there are few labeled neurons with axons descending through the compressed region to the RDA application site. (TIF) [file pone.0071701.s001.tif]

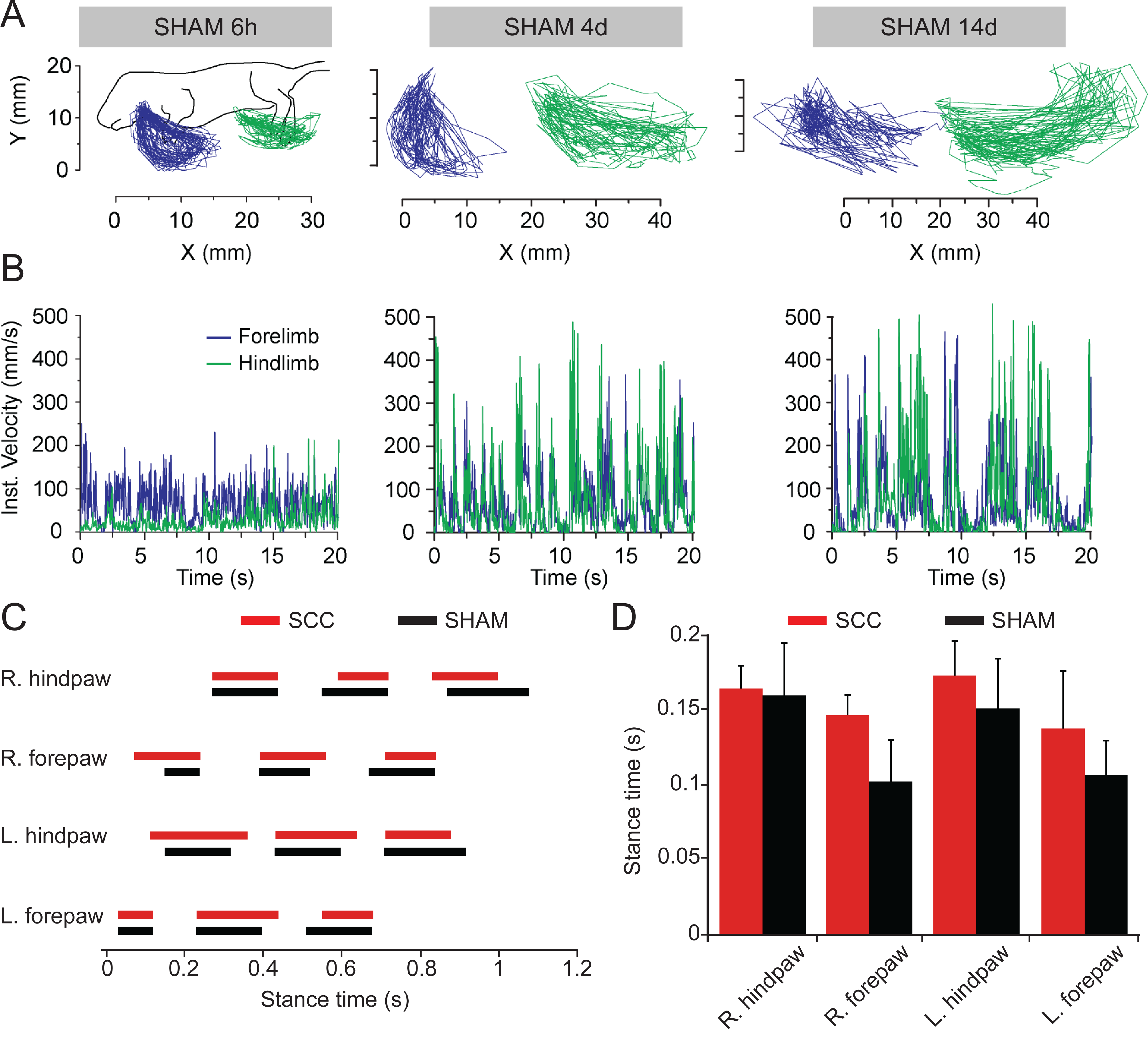

Supplement: Figure S2 — Developmental profile of air stepping in sham control mice, and gait analysis of sham and SCC mice. (A) Kinematic assessment of the trajectories (A) and instantaneous velocities (B) of forepaws (blue traces) and hindpaws (green traces) during air stepping by sham mice at 3 times after surgery. (C and D) Gait analyses comparing sham and SCC mice 24 days after surgery/injury. (C) Representative stance durations of the 4 paws of one SCC mouse (red) and one sham control mouse (black) during voluntary walking along a track. (D) Average stance durations of the 4 paws of SCC and sham mice during single track locomotion. (TIF) [file pone.0071701.s002.tif]

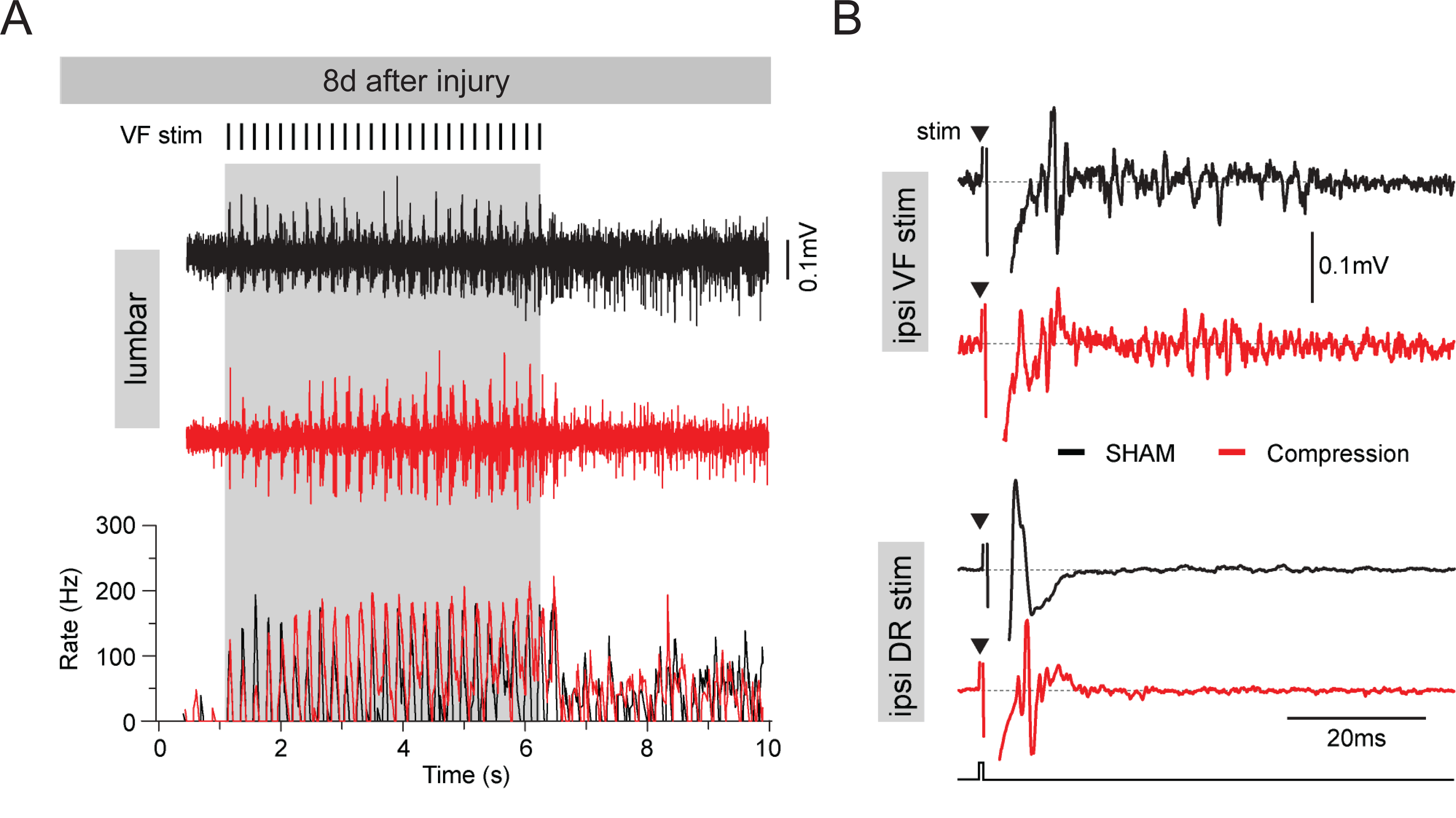

Supplement: Figure S3 — Electrophysiological activity in descending motor pathways 8 days after surgery/injury. (A) Activity pattern in lumbar ventral roots in response to electrical ventral funiculus (VF) train stimulation (25 pulses during 5 s, pulse duration 0.2 ms, x2T–x3T, where T = threshold for generating a response) in one sham control mouse (top black trace) and one SCC mouse (bottom red trace). Bottom plots in A show the instantaneous firing frequency. (B) Activity pattern in lumbar ventral roots in response to electrical single pulse stimulation of the ipsilateral ventral funiculus (ipsi VF, top pair of traces) and ipsilateral dorsal root in the same segment (ipsi DR, bottom pair of traces) in one sham control mouse (black traces) and one SCC mouse (red traces). (TIF) [file pone.0071701.s003.tif]
